# Supplementary figures and images for: Cycloastragenol: A Novel Senolytic Agent That Induces Senescent Cell Apoptosis and Restores Physical Function in TBI-Aged Mice
Source: Int J Mol Sci. 2023 Mar 31;24(7):6554. doi: 10.3390/ijms24076554 (PMC10095196; doi:10.3390/ijms24076554)

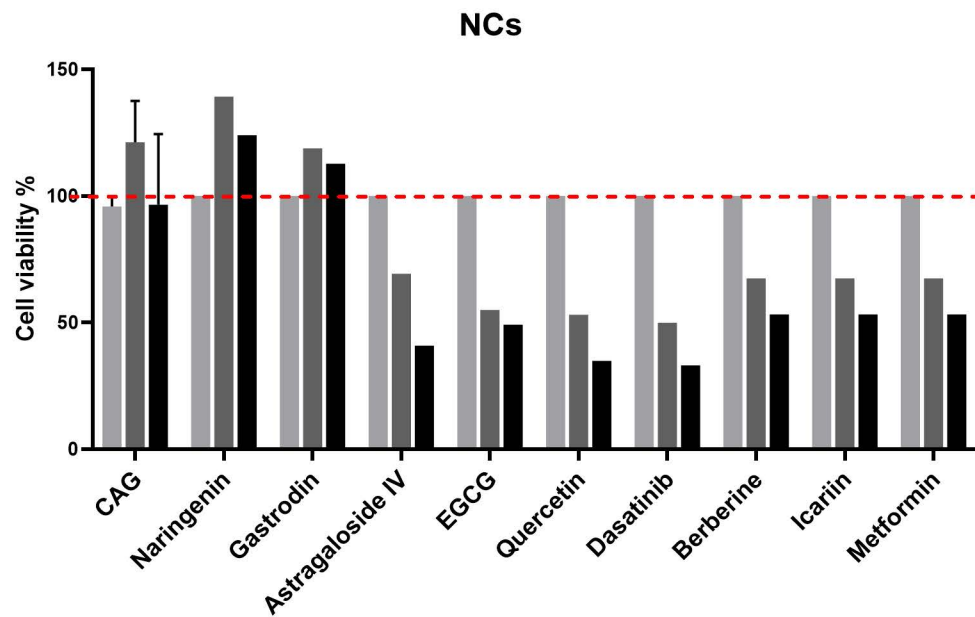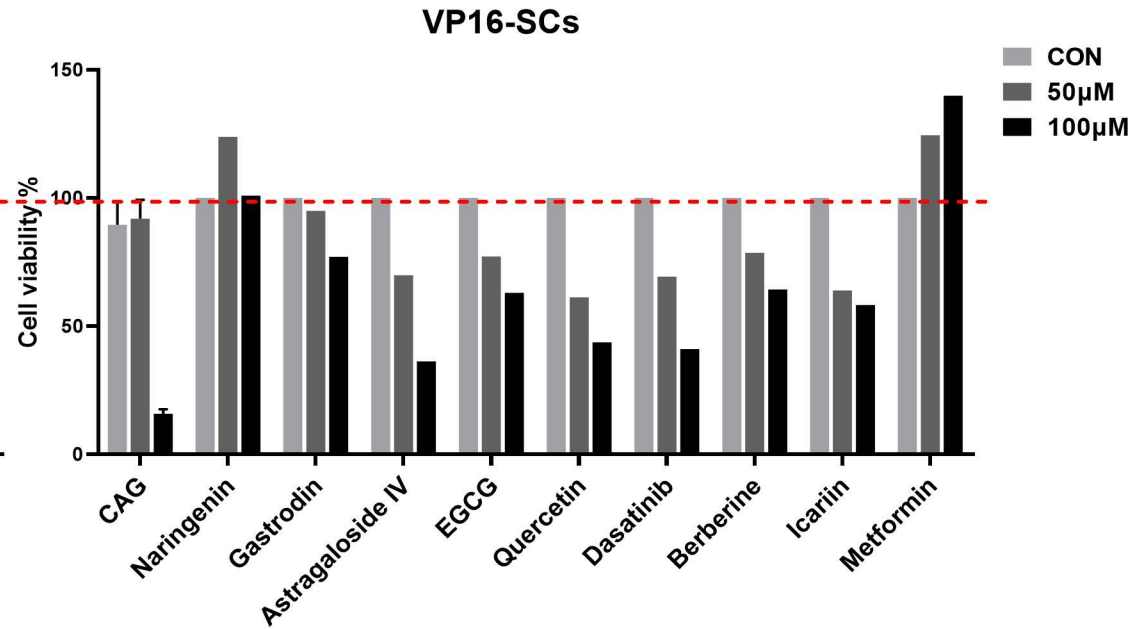

Supplement: Supplementary file 1 [file ijms-24-06554-s001.zip › Supplementary data Figure S1.pdf]
